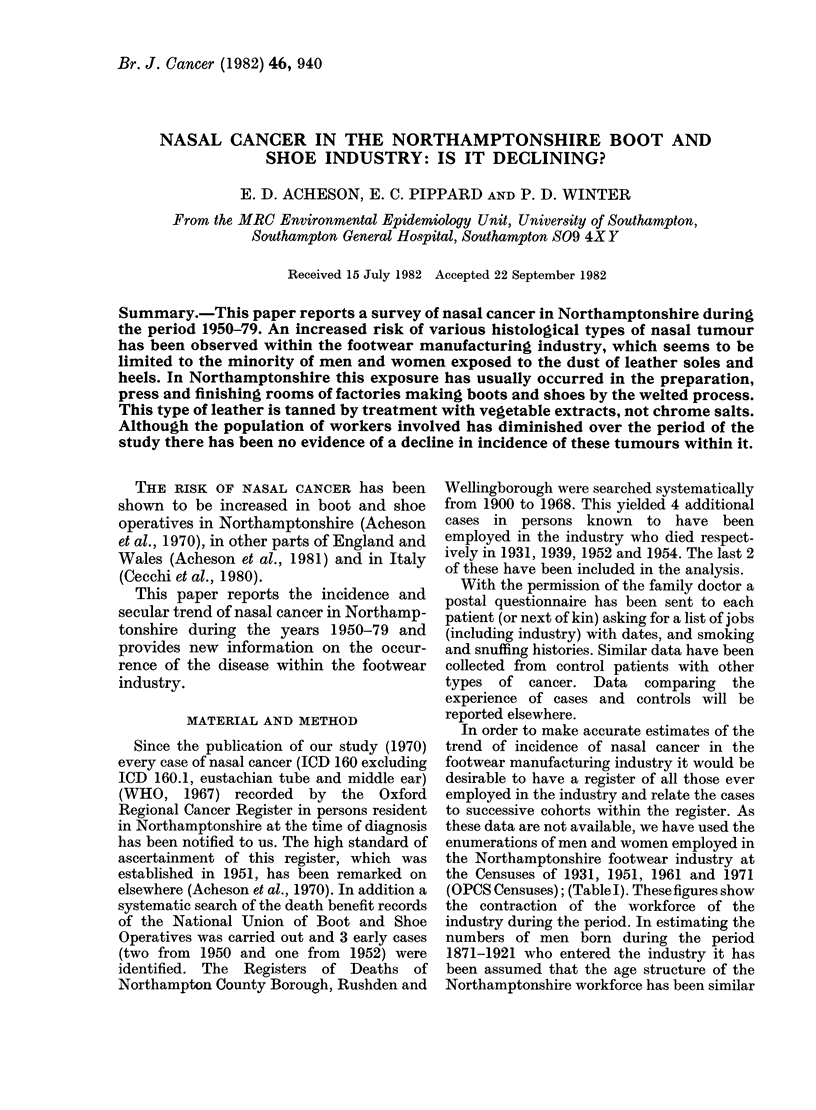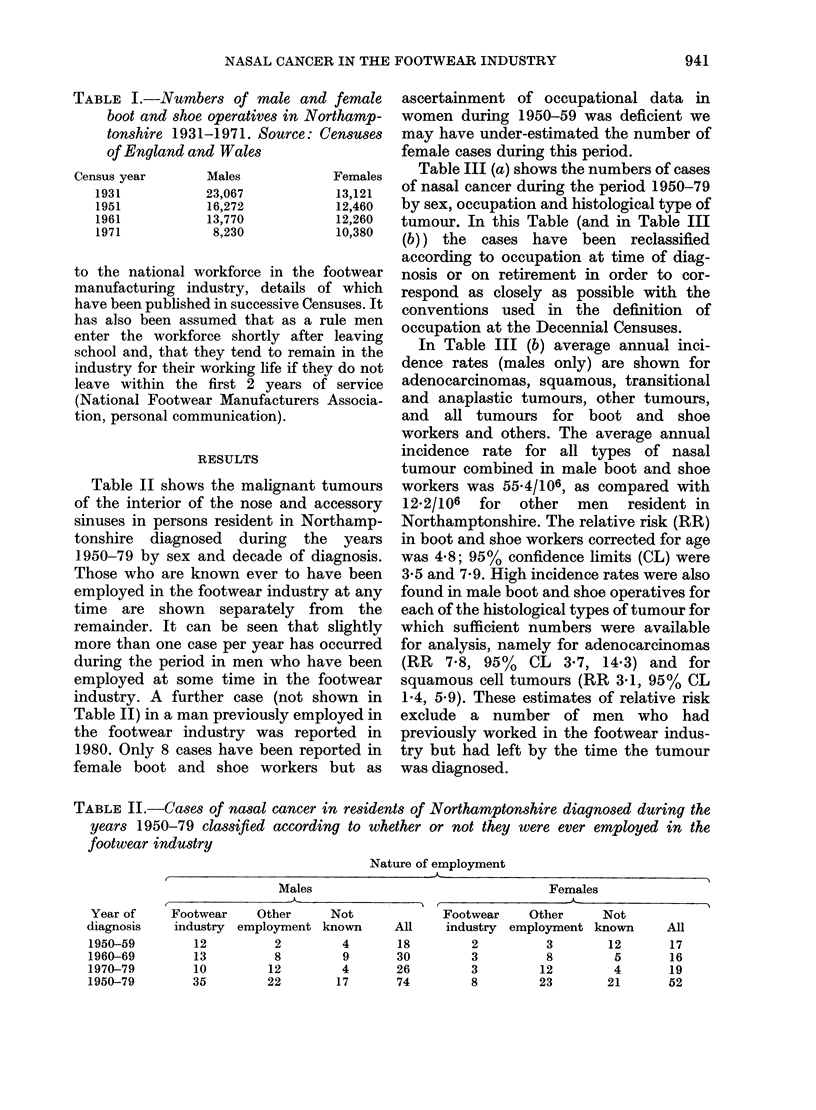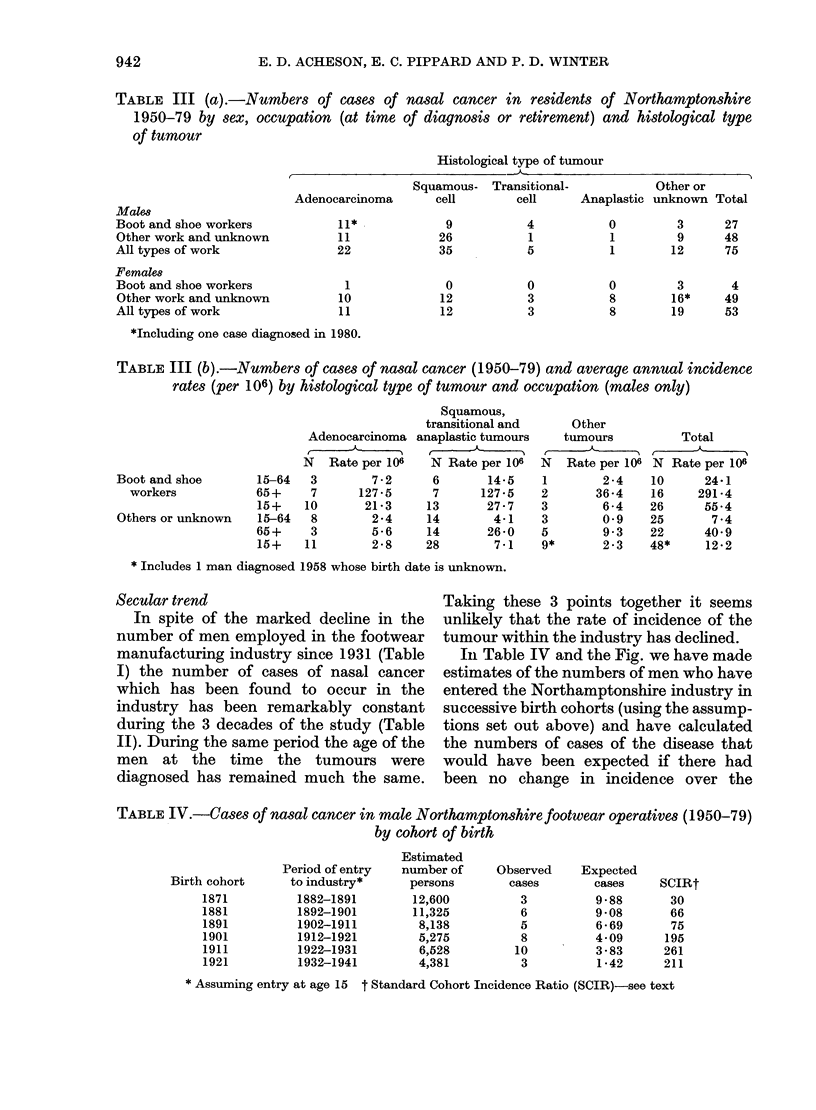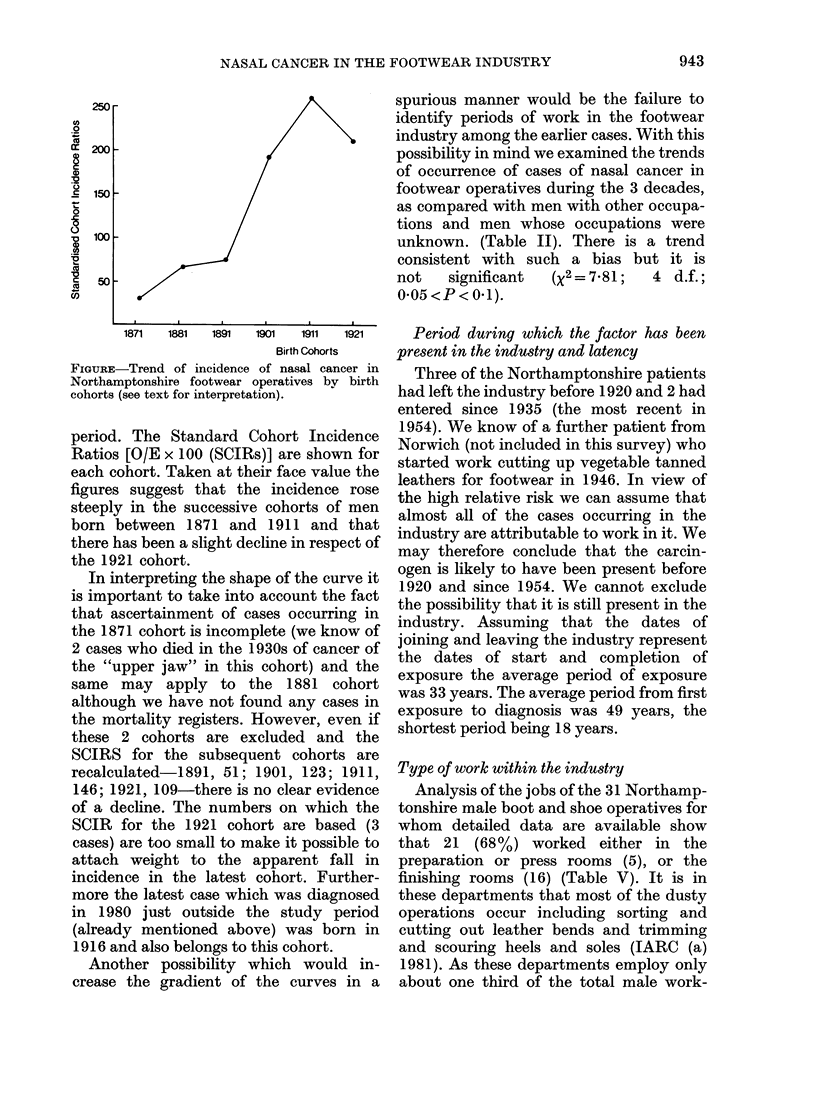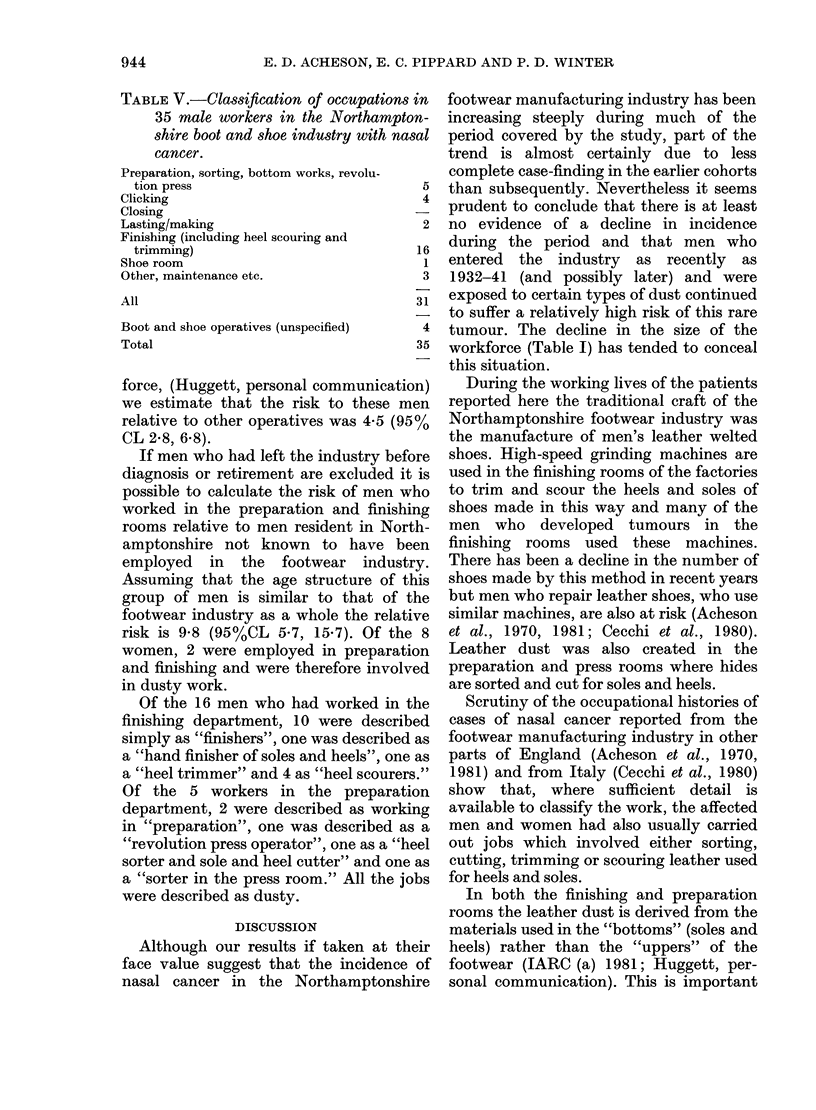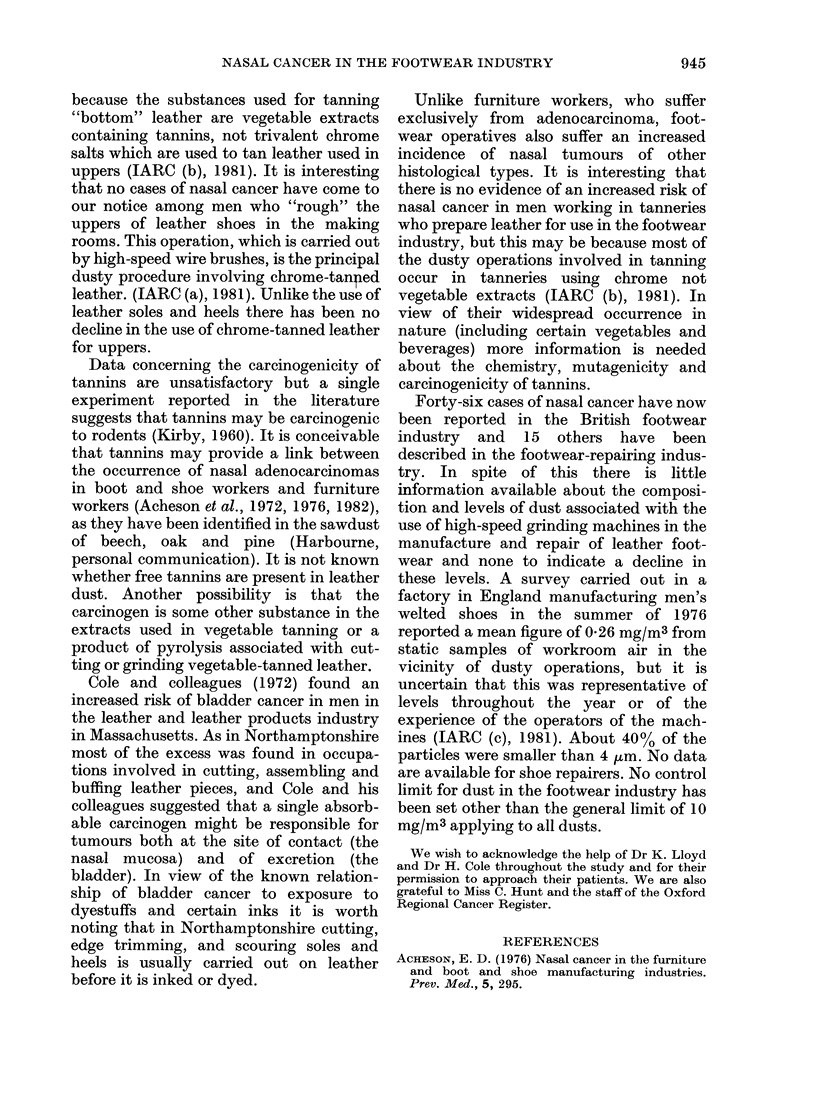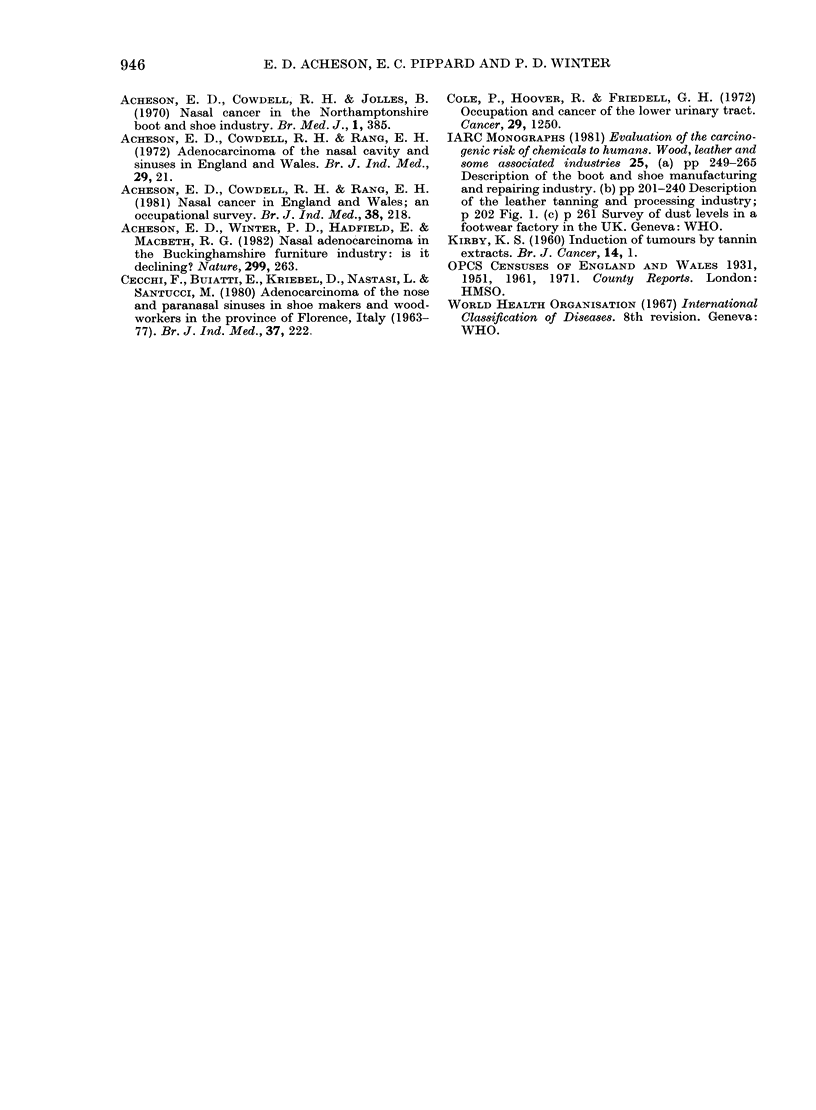# Nasal cancer in the Northamptonshire boot and shoe industry: is it declining?

**DOI:** 10.1038/bjc.1982.305

**Published:** 1982-12

**Authors:** E. D. Acheson, E. C. Pippard, P. D. Winter

## Abstract

This paper reports a survey of nasal cancer in Northamptonshire during the period 1950-79. An increased risk of various histological types of nasal tumour has been observed within the footwear manufacturing industry, which seems to be limited to the minority of men and women exposed to the dust of leather soles and heels. In Northamptonshire this exposure has usually occurred in the preparation, press and finishing rooms of factories making boots and shoes by the welted process. This type of leather is tanned by treatment with vegetable extracts, not chrome salts. Although the population of workers involved has diminished over the period of the study there has been no evidence of a decline in incidence of these tumours within it.


*Br. J. Cancer* (1982) **46**, 940

### NASAL CANCER IN THE NORTHAMPTONSHIRE BOOT AND SHOE INDUSTRY: IS IT DECLINING?

E. D. ACHESON, E. C. PIPPARD AND P. D. WINTER

*From the MRC Environmental Epidemiology Unit, University of Southampton, Southampton General Hospital, Southampton SO9 4XY*

Received 15 July 1982 Accepted 22 September 1982

**Summary.—This paper reports a survey of nasal cancer in Northamptonshire during the period 1950–79. An increased risk of various histological types of nasal tumour has been observed within the footwear manufacturing industry, which seems to be limited to the minority of men and women exposed to the dust of leather soles and heels. In Northamptonshire this exposure has usually occurred in the preparation, press and finishing rooms of factories making boots and shoes by the welted process. This type of leather is tanned by treatment with vegetable extracts, not chrome salts. Although the population of workers involved has diminished over the period of the study there has been no evidence of a decline in incidence of these tumours within it.**

THE RISK OF NASAL CANCER has been shown to be increased in boot and shoe operatives in Northamptonshire (Acheson *et al.*, 1970), in other parts of England and Wales (Acheson *et al.*, 1981) and in Italy (Cecchi *et al.*, 1980).

This paper reports the incidence and secular trend of nasal cancer in Northamptonshire during the years 1950–79 and provides new information on the occurrence of the disease within the footwear industry.

### MATERIAL AND METHOD

Since the publication of our study (1970) every case of nasal cancer (ICD 160 excluding ICD 160.1, eustachian tube and middle ear) (WHO, 1967) recorded by the Oxford Regional Cancer Register in persons resident in Northamptonshire at the time of diagnosis has been notified to us. The high standard of ascertainment of this register, which was established in 1951, has been remarked on elsewhere (Acheson *et al.*, 1970). In addition a systematic search of the death benefit records of the National Union of Boot and Shoe Operatives was carried out and 3 early cases (two from 1950 and one from 1952) were identified. The Registers of Deaths of Northampton County Borough, Rushden and Wellingborough were searched systematically from 1900 to 1968. This yielded 4 additional cases in persons known to have been employed in the industry who died respectively in 1931, 1939, 1952 and 1954. The last 2 of these have been included in the analysis.

With the permission of the family doctor a postal questionnaire has been sent to each patient (or next of kin) asking for a list of jobs (including industry) with dates, and smoking and snuffing histories. Similar data have been collected from control patients with other types of cancer. Data comparing the experience of cases and controls will be reported elsewhere.

In order to make accurate estimates of the trend of incidence of nasal cancer in the footwear manufacturing industry it would be desirable to have a register of all those ever employed in the industry and relate the cases to successive cohorts within the register. As these data are not available, we have used the enumerations of men and women employed in the Northamptonshire footwear industry at the Censuses of 1931, 1951, 1961 and 1971 (OPCS Censuses); (Table I). These figures show the contraction of the workforce of the industry during the period. In estimating the numbers of men born during the period 1871–1921 who entered the industry it has been assumed that the age structure of the Northamptonshire workforce has been similar

TABLE I.—*Numbers of male and female boot and shoe operatives in Northamptonshire* 1931–1971. *Source: Censuses of England and Wales*

| Census year | Males | Females |
|---|---|---|
| 1931 | 23,067 | 13,121 |
| 1951 | 16,272 | 12,460 |
| 1961 | 13,770 | 12,260 |
| 1971 | 8,230 | 10,380 |

to the national workforce in the footwear manufacturing industry, details of which have been published in successive Censuses. It has also been assumed that as a rule men enter the workforce shortly after leaving school and, that they tend to remain in the industry for their working life if they do not leave within the first 2 years of service (National Footwear Manufacturers Association, personal communication).

### RESULTS

Table II shows the malignant tumours of the interior of the nose and accessory sinuses in persons resident in Northamptonshire diagnosed during the years 1950–79 by sex and decade of diagnosis. Those who are known ever to have been employed in the footwear industry at any time are shown separately from the remainder. It can be seen that slightly more than one case per year has occurred during the period in men who have been employed at some time in the footwear industry. A further case (not shown in Table II) in a man previously employed in the footwear industry was reported in 1980. Only 8 cases have been reported in female boot and shoe workers but as ascertainment of occupational data in women during 1950–59 was deficient we may have under-estimated the number of female cases during this period.

Table III (*a*) shows the numbers of cases of nasal cancer during the period 1950–79 by sex, occupation and histological type of tumour. In this Table (and in Table III (*b*)) the cases have been reclassified according to occupation at time of diagnosis or on retirement in order to correspond as closely as possible with the conventions used in the definition of occupation at the Decennial Censuses.

In Table III (*b*) average annual incidence rates (males only) are shown for adenocarcinomas, squamous, transitional and anaplastic tumours, other tumours, and all tumours for boot and shoe workers and others. The average annual incidence rate for all types of nasal tumour combined in male boot and shoe workers was $55{\cdot}4/10^6$, as compared with $12{\cdot}2/10^6$ for other men resident in Northamptonshire. The relative risk (RR) in boot and shoe workers corrected for age was 4·8; 95% confidence limits (CL) were 3·5 and 7·9. High incidence rates were also found in male boot and shoe operatives for each of the histological types of tumour for which sufficient numbers were available for analysis, namely for adenocarcinomas (RR 7·8, 95% CL 3·7, 14·3) and for squamous cell tumours (RR 3·1, 95% CL 1·4, 5·9). These estimates of relative risk exclude a number of men who had previously worked in the footwear industry but had left by the time the tumour was diagnosed.

TABLE II.—*Cases of nasal cancer in residents of Northamptonshire diagnosed during the years* 1950–79 *classified according to whether or not they were ever employed in the footwear industry*

| | Nature of employment | | | | | | | |
|---|---|---|---|---|---|---|---|---|
| | Males | | | | Females | | | |
| Year of diagnosis | Footwear industry | Other employment | Not known | All | Footwear industry | Other employment | Not known | All |
| 1950–59 | 12 | 2 | 4 | 18 | 2 | 3 | 12 | 17 |
| 1960–69 | 13 | 8 | 9 | 30 | 3 | 8 | 5 | 16 |
| 1970–79 | 10 | 12 | 4 | 26 | 3 | 12 | 4 | 19 |
| 1950–79 | 35 | 22 | 17 | 74 | 8 | 23 | 21 | 52 |

TABLE III (*a*).—*Numbers of cases of nasal cancer in residents of Northamptonshire 1950–79 by sex, occupation (at time of diagnosis or retirement) and histological type of tumour*

| | Histological type of tumour | | | | | |
|---|---|---|---|---|---|---|
| | Adenocarcinoma | Squamous-cell | Transitional-cell | Anaplastic | Other or unknown | Total |
| *Males* | | | | | | |
| Boot and shoe workers | 11* | 9 | 4 | 0 | 3 | 27 |
| Other work and unknown | 11 | 26 | 1 | 1 | 9 | 48 |
| All types of work | 22 | 35 | 5 | 1 | 12 | 75 |
| *Females* | | | | | | |
| Boot and shoe workers | 1 | 0 | 0 | 0 | 3 | 4 |
| Other work and unknown | 10 | 12 | 3 | 8 | 16* | 49 |
| All types of work | 11 | 12 | 3 | 8 | 19 | 53 |

*Including one case diagnosed in 1980.

TABLE III (*b*).—*Numbers of cases of nasal cancer* (1950–79) *and average annual incidence rates* (*per* $10^6$) *by histological type of tumour and occupation* (*males only*)

| | | Adenocarcinoma | | Squamous, transitional and anaplastic tumours | | Other tumours | | Total | |
|---|---|---|---|---|---|---|---|---|---|
| | | N | Rate per $10^6$ | N | Rate per $10^6$ | N | Rate per $10^6$ | N | Rate per $10^6$ |
| Boot and shoe | 15–64 | 3 | 7·2 | 6 | 14·5 | 1 | 2·4 | 10 | 24·1 |
| workers | 65+ | 7 | 127·5 | 7 | 127·5 | 2 | 36·4 | 16 | 291·4 |
| | 15+ | 10 | 21·3 | 13 | 27·7 | 3 | 6·4 | 26 | 55·4 |
| Others or unknown | 15–64 | 8 | 2·4 | 14 | 4·1 | 3 | 0·9 | 25 | 7·4 |
| | 65+ | 3 | 5·6 | 14 | 26·0 | 5 | 9·3 | 22 | 40·9 |
| | 15+ | 11 | 2·8 | 28 | 7·1 | 9* | 2·3 | 48* | 12·2 |

* Includes 1 man diagnosed 1958 whose birth date is unknown.

*Secular trend*

In spite of the marked decline in the number of men employed in the footwear manufacturing industry since 1931 (Table I) the number of cases of nasal cancer which has been found to occur in the industry has been remarkably constant during the 3 decades of the study (Table II). During the same period the age of the men at the time the tumours were diagnosed has remained much the same. Taking these 3 points together it seems unlikely that the rate of incidence of the tumour within the industry has declined.

In Table IV and the Fig. we have made estimates of the numbers of men who have entered the Northamptonshire industry in successive birth cohorts (using the assumptions set out above) and have calculated the numbers of cases of the disease that would have been expected if there had been no change in incidence over the

TABLE IV.—*Cases of nasal cancer in male Northamptonshire footwear operatives* (1950–79) *by cohort of birth*

| Birth cohort | Period of entry to industry* | Estimated number of persons | Observed cases | Expected cases | SCIR† |
|---|---|---|---|---|---|
| 1871 | 1882–1891 | 12,600 | 3 | 9·88 | 30 |
| 1881 | 1892–1901 | 11,325 | 6 | 9·08 | 66 |
| 1891 | 1902–1911 | 8,138 | 5 | 6·69 | 75 |
| 1901 | 1912–1921 | 5,275 | 8 | 4·09 | 195 |
| 1911 | 1922–1931 | 6,528 | 10 | 3·83 | 261 |
| 1921 | 1932–1941 | 4,381 | 3 | 1·42 | 211 |

* Assuming entry at age 15 † Standard Cohort Incidence Ratio (SCIR)—see text

FIGURE—Trend of incidence of nasal cancer in Northamptonshire footwear operatives by birth cohorts (see text for interpretation).

period. The Standard Cohort Incidence Ratios [O/E × 100 (SCIRs)] are shown for each cohort. Taken at their face value the figures suggest that the incidence rose steeply in the successive cohorts of men born between 1871 and 1911 and that there has been a slight decline in respect of the 1921 cohort.

In interpreting the shape of the curve it is important to take into account the fact that ascertainment of cases occurring in the 1871 cohort is incomplete (we know of 2 cases who died in the 1930s of cancer of the "upper jaw" in this cohort) and the same may apply to the 1881 cohort although we have not found any cases in the mortality registers. However, even if these 2 cohorts are excluded and the SCIRS for the subsequent cohorts are recalculated—1891, 51; 1901, 123; 1911, 146; 1921, 109—there is no clear evidence of a decline. The numbers on which the SCIR for the 1921 cohort are based (3 cases) are too small to make it possible to attach weight to the apparent fall in incidence in the latest cohort. Furthermore the latest case which was diagnosed in 1980 just outside the study period (already mentioned above) was born in 1916 and also belongs to this cohort.

Another possibility which would increase the gradient of the curves in a spurious manner would be the failure to identify periods of work in the footwear industry among the earlier cases. With this possibility in mind we examined the trends of occurrence of cases of nasal cancer in footwear operatives during the 3 decades, as compared with men with other occupations and men whose occupations were unknown. (Table II). There is a trend consistent with such a bias but it is not significant ($\chi^2 = 7{\cdot}81$; 4 d.f.; $0{\cdot}05 < P < 0{\cdot}1$).

*Period during which the factor has been present in the industry and latency*

Three of the Northamptonshire patients had left the industry before 1920 and 2 had entered since 1935 (the most recent in 1954). We know of a further patient from Norwich (not included in this survey) who started work cutting up vegetable tanned leathers for footwear in 1946. In view of the high relative risk we can assume that almost all of the cases occurring in the industry are attributable to work in it. We may therefore conclude that the carcinogen is likely to have been present before 1920 and since 1954. We cannot exclude the possibility that it is still present in the industry. Assuming that the dates of joining and leaving the industry represent the dates of start and completion of exposure the average period of exposure was 33 years. The average period from first exposure to diagnosis was 49 years, the shortest period being 18 years.

*Type of work within the industry*

Analysis of the jobs of the 31 Northamptonshire male boot and shoe operatives for whom detailed data are available show that 21 (68%) worked either in the preparation or press rooms (5), or the finishing rooms (16) (Table V). It is in these departments that most of the dusty operations occur including sorting and cutting out leather bends and trimming and scouring heels and soles (IARC (a) 1981). As these departments employ only about one third of the total male work-

TABLE V.—*Classification of occupations in 35 male workers in the Northamptonshire boot and shoe industry with nasal cancer.*

| | |
|---|---|
| Preparation, sorting, bottom works, revolution press | 5 |
| Clicking | 4 |
| Closing | — |
| Lasting/making | 2 |
| Finishing (including heel scouring and trimming) | 16 |
| Shoe room | 1 |
| Other, maintenance etc. | 3 |
| All | 31 |
| Boot and shoe operatives (unspecified) | 4 |
| Total | 35 |

force, (Huggett, personal communication) we estimate that the risk to these men relative to other operatives was 4·5 (95% CL 2·8, 6·8).

If men who had left the industry before diagnosis or retirement are excluded it is possible to calculate the risk of men who worked in the preparation and finishing rooms relative to men resident in Northamptonshire not known to have been employed in the footwear industry. Assuming that the age structure of this group of men is similar to that of the footwear industry as a whole the relative risk is 9·8 (95%CL 5·7, 15·7). Of the 8 women, 2 were employed in preparation and finishing and were therefore involved in dusty work.

Of the 16 men who had worked in the finishing department, 10 were described simply as "finishers", one was described as a "hand finisher of soles and heels", one as a "heel trimmer" and 4 as "heel scourers." Of the 5 workers in the preparation department, 2 were described as working in "preparation", one was described as a "revolution press operator", one as a "heel sorter and sole and heel cutter" and one as a "sorter in the press room." All the jobs were described as dusty.

### DISCUSSION

Although our results if taken at their face value suggest that the incidence of nasal cancer in the Northamptonshire footwear manufacturing industry has been increasing steeply during much of the period covered by the study, part of the trend is almost certainly due to less complete case-finding in the earlier cohorts than subsequently. Nevertheless it seems prudent to conclude that there is at least no evidence of a decline in incidence during the period and that men who entered the industry as recently as 1932–41 (and possibly later) and were exposed to certain types of dust continued to suffer a relatively high risk of this rare tumour. The decline in the size of the workforce (Table I) has tended to conceal this situation.

During the working lives of the patients reported here the traditional craft of the Northamptonshire footwear industry was the manufacture of men's leather welted shoes. High-speed grinding machines are used in the finishing rooms of the factories to trim and scour the heels and soles of shoes made in this way and many of the men who developed tumours in the finishing rooms used these machines. There has been a decline in the number of shoes made by this method in recent years but men who repair leather shoes, who use similar machines, are also at risk (Acheson *et al.*, 1970, 1981; Cecchi *et al.*, 1980). Leather dust was also created in the preparation and press rooms where hides are sorted and cut for soles and heels.

Scrutiny of the occupational histories of cases of nasal cancer reported from the footwear manufacturing industry in other parts of England (Acheson *et al.*, 1970, 1981) and from Italy (Cecchi *et al.*, 1980) show that, where sufficient detail is available to classify the work, the affected men and women had also usually carried out jobs which involved either sorting, cutting, trimming or scouring leather used for heels and soles.

In both the finishing and preparation rooms the leather dust is derived from the materials used in the "bottoms" (soles and heels) rather than the "uppers" of the footwear (IARC (a) 1981; Huggett, personal communication). This is important because the substances used for tanning "bottom" leather are vegetable extracts containing tannins, not trivalent chrome salts which are used to tan leather used in uppers (IARC (b), 1981). It is interesting that no cases of nasal cancer have come to our notice among men who "rough" the uppers of leather shoes in the making rooms. This operation, which is carried out by high-speed wire brushes, is the principal dusty procedure involving chrome-tanned leather. (IARC (a), 1981). Unlike the use of leather soles and heels there has been no decline in the use of chrome-tanned leather for uppers.

Data concerning the carcinogenicity of tannins are unsatisfactory but a single experiment reported in the literature suggests that tannins may be carcinogenic to rodents (Kirby, 1960). It is conceivable that tannins may provide a link between the occurrence of nasal adenocarcinomas in boot and shoe workers and furniture workers (Acheson *et al.*, 1972, 1976, 1982), as they have been identified in the sawdust of beech, oak and pine (Harbourne, personal communication). It is not known whether free tannins are present in leather dust. Another possibility is that the carcinogen is some other substance in the extracts used in vegetable tanning or a product of pyrolysis associated with cutting or grinding vegetable-tanned leather.

Cole and colleagues (1972) found an increased risk of bladder cancer in men in the leather and leather products industry in Massachusetts. As in Northamptonshire most of the excess was found in occupations involved in cutting, assembling and buffing leather pieces, and Cole and his colleagues suggested that a single absorbable carcinogen might be responsible for tumours both at the site of contact (the nasal mucosa) and of excretion (the bladder). In view of the known relationship of bladder cancer to exposure to dyestuffs and certain inks it is worth noting that in Northamptonshire cutting, edge trimming, and scouring soles and heels is usually carried out on leather before it is inked or dyed.

Unlike furniture workers, who suffer exclusively from adenocarcinoma, footwear operatives also suffer an increased incidence of nasal tumours of other histological types. It is interesting that there is no evidence of an increased risk of nasal cancer in men working in tanneries who prepare leather for use in the footwear industry, but this may be because most of the dusty operations involved in tanning occur in tanneries using chrome not vegetable extracts (IARC (b), 1981). In view of their widespread occurrence in nature (including certain vegetables and beverages) more information is needed about the chemistry, mutagenicity and carcinogenicity of tannins.

Forty-six cases of nasal cancer have now been reported in the British footwear industry and 15 others have been described in the footwear-repairing industry. In spite of this there is little information available about the composition and levels of dust associated with the use of high-speed grinding machines in the manufacture and repair of leather footwear and none to indicate a decline in these levels. A survey carried out in a factory in England manufacturing men's welted shoes in the summer of 1976 reported a mean figure of 0·26 $mg/m^3$ from static samples of workroom air in the vicinity of dusty operations, but it is uncertain that this was representative of levels throughout the year or of the experience of the operators of the machines (IARC (c), 1981). About 40% of the particles were smaller than 4 $\mu m$. No data are available for shoe repairers. No control limit for dust in the footwear industry has been set other than the general limit of 10 $mg/m^3$ applying to all dusts.

We wish to acknowledge the help of Dr K. Lloyd and Dr H. Cole throughout the study and for their permission to approach their patients. We are also grateful to Miss C. Hunt and the staff of the Oxford Regional Cancer Register.

### REFERENCES

ACHESON, E. D. (1976) Nasal cancer in the furniture and boot and shoe manufacturing industries. *Prev. Med.*, **5**, 295.

ACHESON, E. D., COWDELL, R. H. & JOLLES, B. (1970) Nasal cancer in the Northamptonshire boot and shoe industry. *Br. Med. J.*, **1**, 385.

ACHESON, E. D., COWDELL, R. H. & RANG, E. H. (1972) Adenocarcinoma of the nasal cavity and sinuses in England and Wales. *Br. J. Ind. Med.*, **29**, 21.

ACHESON, E. D., COWDELL, R. H. & RANG, E. H. (1981) Nasal cancer in England and Wales; an occupational survey. *Br. J. Ind. Med.*, **38**, 218.

ACHESON, E. D., WINTER, P. D., HADFIELD, E. & MACBETH, R. G. (1982) Nasal adenocarcinoma in the Buckinghamshire furniture industry: is it declining? *Nature*, **299**, 263.

CECCHI, F., BUIATTI, E., KRIEBEL, D., NASTASI, L. & SANTUCCI, M. (1980) Adenocarcinoma of the nose and paranasal sinuses in shoe makers and woodworkers in the province of Florence, Italy (1963–77). *Br. J. Ind. Med.*, **37**, 222.

COLE, P., HOOVER, R. & FRIEDELL, G. H. (1972) Occupation and cancer of the lower urinary tract. *Cancer*, **29**, 1250.

IARC MONOGRAPHS (1981) *Evaluation of the carcinogenic risk of chemicals to humans. Wood, leather and some associated industries* **25**, (a) pp 249–265 Description of the boot and shoe manufacturing and repairing industry. (b) pp 201–240 Description of the leather tanning and processing industry; p 202 Fig. 1. (c) p 261 Survey of dust levels in a footwear factory in the UK. Geneva: WHO.

KIRBY, K. S. (1960) Induction of tumours by tannin extracts. *Br. J. Cancer*, **14**, 1.

OPCS CENSUSES OF ENGLAND AND WALES 1931, 1951, 1961, 1971. *County Reports*. London: HMSO.

WORLD HEALTH ORGANISATION (1967) *International Classification of Diseases*. 8th revision. Geneva: WHO.